# Cannabis and Multiple Sclerosis—The Way Forward

**DOI:** 10.3389/fneur.2017.00299

**Published:** 2017-06-23

**Authors:** Thorsten Rudroff, Justin M. Honce

**Affiliations:** ^1^Department of Health and Exercise Science, Colorado State University, Fort Collins, CO, United States; ^2^Department of Radiology, University of Colorado School of Medicine, Aurora, CO, United States

**Keywords:** cannabis, multiple sclerosis, marijuana, observational research, National Institutes of Health (U.S.)

The cannabis plant contains up to 110 cannabinoids, though Δ^9^-tetrahydrocannabinol (THC) and cannabidiol (CBD) have been the primary focus of medical investigation. THC has psychoactive effects, such as cognitive impairments, psychosis, dysphoria, and anxiety, whereas CBD is non-intoxicating and has anti-inflammatory, analgesic, and antipsychotic properties, and can counter several unwanted side effects of THC.

A significant number of people with multiple sclerosis (PwMS) are using or considering using cannabis for a range of symptoms. Recent studies have indicated that there is a wide acceptance of cannabis within the MS community, with 20–60% of PwMS currently using cannabis, and 50–90% would consider usage if it were legal and more scientific evidence was available (1–3). Twenty-eight states and Washington, DC currently have laws legalizing cannabis use for a variety of medical conditions. An additional 16 states have passed laws that specifically permit the medical use of CBD. Cannabis advocates believe there is a chance at least 11 more states will legalize cannabis for medical use in the near term. Therefore, the number of PwMS using cannabis to treat their symptoms is expected to rise.

As was made clear during a recent meeting sponsored by the National Institutes of Health (NIH), *Marijuana and Cannabinoids: A Neuroscience Research Summit*, there are many uncertainties about the positive and negative effects associated with cannabis use in PwMS, However, it is known that cannabis strains, containing CBD levels equal or higher than THC, have positive effects on muscle spasticity (4) and pain in PwMS (5). These positive effects of cannabis on spasticity and pain and its safety have also been emphasized by the American Academy of Neurology (6).

It is obvious that much more research is needed to investigate the effects of cannabis in PwMS. Unanswered questions which need further research include: Which MS related symptom or symptoms are best treated with cannabis? Which constituent synthetic or plant-derived cannabinoid best treats MS? Does using a combination of cannabinoids increase efficacy, and if so, do different ratios provide different results? Does the dosage and route of cannabis administration (smoking, edibles, drops, oromucosal spray, etc.) impact the efficacy of cannabis? Other intriguing research questions include: Does the effects of cannabis on motor and cognitive function persist after cessation of cannabis use? How does cannabis interact with other commonly used MS drugs? And crucially, do cannabinoids have therapeutic effects that extend beyond symptom relief, such as immunomodulatory or neuroprotective effects?

To answer these questions, there needs to be a substantial increase in the number of research grants for the study of cannabis use in MS. However, although the American Academy of Neurology states that cannabis is effective for the treatment of pain and spasticity, and a recent online survey showed that approximately 50% of PwMS already use cannabis without a medical card and any scientific guidance (3), organizations such as the National MS Society and NIH are hesitant to support further research into the *benefits* of cannabis in MS.

The National Institute of Drug Abuse (NIDA) funds more than 90% of all research on many of the most commonly used recreational drugs. Its mission “is to lead the nation in bringing the power of science to bear on drug *abuse and addiction*.” Certainly, drugs such as cannabis may produce other effects, including positive ones that have nothing to do with “abuse and addiction.” Unfortunately, this is not part of NIDA’s mission. Scientists in search of research grants from NIDA are well aware of this, as it is mandated that research on controlled substances must be funded by NIDA, even if the proposed goals of the study may be more appropriate for a different institute. As a result, scientists often emphasize the negative effects to get their research funded. The result is that the majority of information on drugs published in the scientific literature and popular press is biased toward the negative aspects of drug use.

It is important to note that countries outside the US often demonstrate a different approach to cannabis research in MS. For example, in the United Kingdom there have been two trials funded by the Medical Research Council (the equivalent of the NIH) to look into the beneficial effects of cannabis in MS, one as symptomatic treatment for spasticity (7), and the other for the reduction of progression (8, 9). Interestingly, the Cannabinoid Use in Progressive Inflammatory brain Disease trial (8, 9) does not rule out possible neuroprotective effects of other cannabinoids administered *via* other routes, and cannabinoid administration with enhanced targeted delivery to the immune system might show immunomodulatory effects.

There are many open questions regarding cannabis use, including optimal strains, frequency of use, other dosage questions, risks of long-term use, and which symptoms it effectively treats. These are all important questions in which the NIH and MS foundations should be interested. Unfortunately, up to date, NIH has not funded research grants on the benefits of cannabis in MS (10). Furthermore, there are no current research projects on cannabis funded by the National MS Society (11). Why is this the case? We hypothesize that while grant reviewers make the argument that randomized controlled trials (RCTs) are needed for this research, few if any are possible in the current legal framework. To investigate the effects of cannabis, a researcher must win approval from the Food and Drug Administration (FDA), Public Health Service, Drug Enforcement Administration (DEA), NIDA, and the Institutional Review Board (IRB). For comparison, to research heroin a researcher must get approval from FDA, DEA, and IRB. Investigators are required to possess a DEA schedule I license in order to perform interventional studies, which is the biggest challenge. Currently, only few laboratories in the United States have a schedule I license. Therefore, it is unrealistic to require cannabis research to be performed in the same manner as traditional medications as long as cannabis remains Schedule I. Instead of RCTs, observational studies would provide initial evidence toward the positive and/or negative effects of cannabis use in PwMS. Observational studies would also serve as the foundation and source of necessary preliminary data for future interventional studies which could be initiated either when the DEA scheduling for cannabis is changed or when MS investigators acquire a Schedule I license. In our opinion, it is important to start to investigate cannabis use in MS with robust observational trials, such as Phase IV studies and other comparative effective research designs. In fact, a recent Cochrane review concluded that largely observational research leads to the same conclusions as RCTs, with very few exceptions (12). However, readers must carefully assess the four possible explanations for associations resulting from observational studies: bias, confounds, chance, and cause (13).

An important current limitation of interventional studies of cannabis is that they can only be performed with government cannabis, grown at a single facility at the University of Mississippi, overseen and under contract by NIDA. Cannabis is the only substance for which NIDA has a monopoly. Unfortunately, these federal products do not reflect the “real-world” cannabis that PwMS currently use. Furthermore, it can take NIDA 20 months to finalize simple strains requested for a study and often they are unable to provide some originally requested strains, easily produced by other US growers (14).

It is important that researcher investigators are able to use higher potency cannabis and more varieties of cannabis than are currently available through NIDA, as these higher potency varieties are frequently used by PwMS. Consequently, although an RCT using government cannabis may be more scientifically rigorous it will lack *external validity*. To assess the effects of cannabis use in PwMS, it is crucial to recruit and investigate the effects in PwMS who are currently using cannabis obtained from local dispensaries. One downside of this approach may be that the cannabis products can be mislabeled, which has been shown by a recent study (15). In this study, cannabis products had significantly more THC than labeled, placing patients at risk of adverse effects, including drug interactions. Furthermore, the FDA gave out several warning letters in 2015 and 2016 about inappropriate and illegal medical claims for CBD formulations manufactured or distributed by US companies. They cited cases where the manufacturer claimed “therapeutically active” concentrations of CBD. However, analytical tests in FDA laboratories found no active ingredient. In addition, there is concern about the presence of fertilizers, pesticides, rodenticides, fungicides, heavy metals, molds, microbes, and other chemical contaminants that may be health risks (16). Therefore, product quality tests and blood analysis at appropriate times after cannabis consumption are mandatory. Another solution to this problem would be that DEA finally take steps to allow other entities with strict quality control practices to supply cannabis for research purposes.

If the issues raised in this manuscript are addressed, these new directions cannabis research may help to achieve the goal for unanimous support of the medicinal use of cannabis for PwMS.

## Author Contributions

TR and JH contributed to drafting the article and revising it critically for important intellectual content. All the authors approved the final version of the manuscript.

## Conflict of Interest Statement

The authors declare that the research was conducted in the absence of any commercial or financial relationships that could be construed as a potential conflict of interest.
